# Application of a Microfluidic Lab-on-a-Chip Platform Coupled with Automated Motility Analysis for Phenotypic Drug Screening of *Trypanosoma cruzi* Trypomastigotes

**DOI:** 10.3390/s26206517

**Published:** 2026-10-14

**Authors:** Daniel A. Chamorro, María Fernanda Cadena Vizuete, Gina Layedra, Ramiro Isa-Jara, Marc A. Giulianotti, Clemencia Pinilla, Maximiliano Pérez, Catalina D. Alba Soto, Maria Laura Belaunzarán, Betiana Lerner, Guadalupe Gimenez

**Affiliations:** 1 Centro de Ingeniería de Recubrimientos Especiales y Nanoestructuras, Universidad Tecnológica Nacional, Buenos Aires B1706EAG, Argentina; daniel.chamorro89@gmail.com (D.A.C.); gina-layedra@hotmail.com (G.L.); ramfer.isaj@gmail.com (R.I.-J.); max@fullgen.com.ar (M.P.); 2 Departamento de Microbiología, Parasitología e Inmunología, Facultad de Medicina, Universidad de Buenos Aires, Buenos Aires C1121A6B, Argentinacatalina.alba@gmail.com (C.D.A.S.); mbelaunzaran@fmed.uba.ar (M.L.B.); 3 Instituto de Investigaciones en Microbiología y Parasitología Médica, Consejo Nacional de Investigaciones Científicas y Técnicas—CONICET, Universidad Buenos Aires, Buenos Aires C1121A6B, Argentina; 4 Facultad de Informática y Electrónica, Escuela Superior Politécnica de Chimborazo, Riobamba 60104, Ecuador; 5 Department of Medicinal Chemistry, Institute for Translational Neuroscience, University of Minnesota, Minneapolis, MN 55455, USA; giuli050@umn.edu (M.A.G.); pinil005@umn.edu (C.P.)

**Keywords:** Lab-on-a-Chip, microfluidics, *Trypanosoma cruzi*, trypomastigotes, phenotypic drug screening, automated image analysis, motility assay, Chagas disease

## Abstract

Chagas disease remains a major neglected tropical disease, highlighting the need for new anti-*Trypanosoma cruzi* compounds and improved approaches for their phenotypic evaluation. Here, we extended a previously developed Lab-on-a-Chip (LOC) platform integrating a serial dilution generator and automated label-free motility analysis (TR_KF) to the direct screening of extracellular *T. cruzi* trypomastigotes. The platform was validated in two steps using benznidazole as the reference drug. First, automated motility quantification in conventional 96-well plates was compared with manual Neubauer counting, yielding comparable concentration–response curves and IC_50_ values. Second, the complete LOC–TR_KF platform was compared with the conventional assay, producing an IC_50_ that was not significantly different from the reference method. The validated platform was subsequently applied to screen ten candidate samples, identifying combinatorial libraries **2161-X** and **2160-X** as the most active against trypomastigotes. Comparison with previously reported intracellular amastigote data revealed marked stage-dependent differences in compound activity, while pre-treatment with both libraries reduced parasite infectivity by approximately 50%. Overall, the integrated LOC–TR_KF platform provides pharmacological responses comparable to conventional assays while reducing assay-solution requirements, manual handling and enabling automated, label-free phenotypic screening of *T. cruzi* trypomastigotes.

## 1. Introduction

Chagas disease is a parasitic infection caused by the protozoan *Trypanosoma cruzi* and transmitted by insect vectors from the subfamily Triatominae. However, transmission also occurs through other routes, including congenital transmission, organ transplantation, blood transfusion, and oral ingestion of food contaminated with infected triatomine feces [1]. The World Health Organization classifies Chagas disease as a neglected tropical disease (NTD), a diverse group of conditions that primarily affect populations living in impoverished communities [2]. More than a century after its discovery, Chagas disease continues to represent a substantial global health burden. Over 7 million individuals are estimated to be infected worldwide, predominantly with chronic infection [2]; however, only a small proportion of infected individuals receive etiological treatment. Originally endemic to Latin America, Chagas disease has also become a public health concern in non-endemic regions, including North America and Europe, largely as a consequence of population migration [3].

Chagas disease progresses through an acute and a chronic phase. The acute phase typically lasts 4–8 weeks following infection and is characterized by high parasitemia due to circulating trypomastigotes. Although frequently asymptomatic, it may present with fever, swelling at the inoculation site, and other nonspecific manifestations. Following the acute phase, most untreated individuals enter the chronic phase, which may remain asymptomatic for prolonged periods. During chronic infection, circulating parasites are scarce, while intracellular amastigotes persist within infected tissues. Approximately 20–30% of chronically infected individuals eventually develop clinical manifestations, including cardiac complications, digestive disorders, or a combination of both [4].

The current therapy for Chagas disease, introduced over five decades ago, relies on monotherapy with nitro-derivative drugs, either nifurtimox or benznidazole (BZN). Although these drugs can be effective, particularly when administered during acute infection, treatment is prolonged and frequently associated with adverse reactions, while therapeutic efficacy is more limited in established chronic disease [5]. In addition, variability in parasite susceptibility to these drugs has been reported. These limitations, together with the restricted investment in drug development for neglected diseases, underscore the need for new antitrypanosomal compounds and improved approaches for their identification [6,7,8].

Phenotypic screening remains a major strategy for antitrypanosomal drug discovery because it evaluates compound activity directly in the biological system without requiring prior knowledge of a specific molecular target [9,10]. Conventional phenotypic assays, however, may require substantial amounts of reagents and parasite material and frequently rely on manual microscopic evaluation, which is labor-intensive and susceptible to operator-dependent variability. Reporter-based and automated image-based approaches have improved the scalability and objectivity of parasite screening [9,11,12]. Nevertheless, reporter-based assays generally require genetically modified parasite lines and appropriate detection systems [13,14,15,16], whereas automated image analysis can provide quantitative phenotypic information directly from microscopy images and reduce reliance on subjective visual assessment [11].

Lab-on-a-Chip (LOC) and other microfluidic technologies have emerged as attractive alternatives for drug screening, enabling assay miniaturization, precise spatiotemporal control of the experimental microenvironment, and parallel analysis of multiple experimental conditions using only a fraction of the reagents and sample volumes required by conventional plate-based assays [17,18,19]. Concentration-gradient generators are particularly attractive for pharmacological applications because they allow the automatic, on-chip generation of serial dilutions, replacing manual pipetting-based dilution series and substantially improving reproducibility, precision, and time efficiency compared with conventional preparation methods [20,21]. Coupling these platforms with automated, image-based analysis further reduces reliance on subjective visual scoring, an approach that has similarly driven the development of label-free, motility- or morphology-based high-content screening methods for other neglected tropical disease pathogens, including *Trypanosoma brucei* and *Schistosoma* spp. [22,23,24]. Microfluidic and lab-on-a-chip platforms have also been applied to *Plasmodium* spp., although mainly for diagnostic purposes [25,26].

Remarkably, compound susceptibility can differ across *T. cruzi* life-cycle stages [27,28]. Intracellular amastigotes and extracellular trypomastigotes represent biologically distinct mammalian stages that differ in their replicative status, cellular environment, and direct accessibility to compounds. Consequently, activity against one parasite stage cannot necessarily be extrapolated to another, and stage-specific assays can provide complementary information on the pharmacological profile of candidate compounds [29,30]. Indeed, recent phenotypic profiling studies have demonstrated marked stage-dependent differences in compound susceptibility, with several inhibitors active against intracellular amastigotes showing substantially reduced or undetectable activity against trypomastigotes [31]. These findings highlight the value of evaluating compound activity across biologically distinct parasite stages, as assays targeting extracellular trypomastigotes can provide pharmacological information complementary to that obtained from intracellular amastigote assays.

In our previous work, we developed and validated a microfluidic LOC platform incorporating a serial dilution generator and automated image analysis for drug screening against intracellular *T. cruzi* amastigotes [32]. In that assay, compound activity against the intracellular stage was assessed indirectly by quantifying the subsequent release of trypomastigotes from infected Vero cells, and the pharmacological results obtained with the LOC platform showed good agreement with those obtained using a conventional flow-cytometry-based assay. The LOC architecture and TR_KF tracking algorithm developed in that study provide the technological basis for the present work. However, direct compound screening against extracellular trypomastigotes using motility as the phenotypic endpoint had not been validated. Extending the platform to this biologically distinct parasite stage could therefore provide complementary information on stage-dependent compound activity while broadening the applicability of the LOC–TR_KF workflow.

In the present study, we extended the application of the previously developed LOC–TR_KF platform to the direct phenotypic evaluation of *T. cruzi* trypomastigotes. We first assessed the agreement between automated TR_KF motility analysis and conventional manual parasite quantification using BZN as the reference drug and subsequently compared the pharmacological performance of the complete LOC–TR_KF platform with that of the conventional 96-well assay. The validated platform was then applied to a panel of candidate samples previously evaluated against intracellular amastigotes, allowing their activity to be compared across two biologically distinct parasite stages. Finally, we evaluated whether pre-treatment of trypomastigotes with selected candidates affected their subsequent ability to establish infection in mammalian host cells.

## 2. Materials and Methods

### 2.1. Microfluidic Device Design and Fabrication

**Figure 1:**
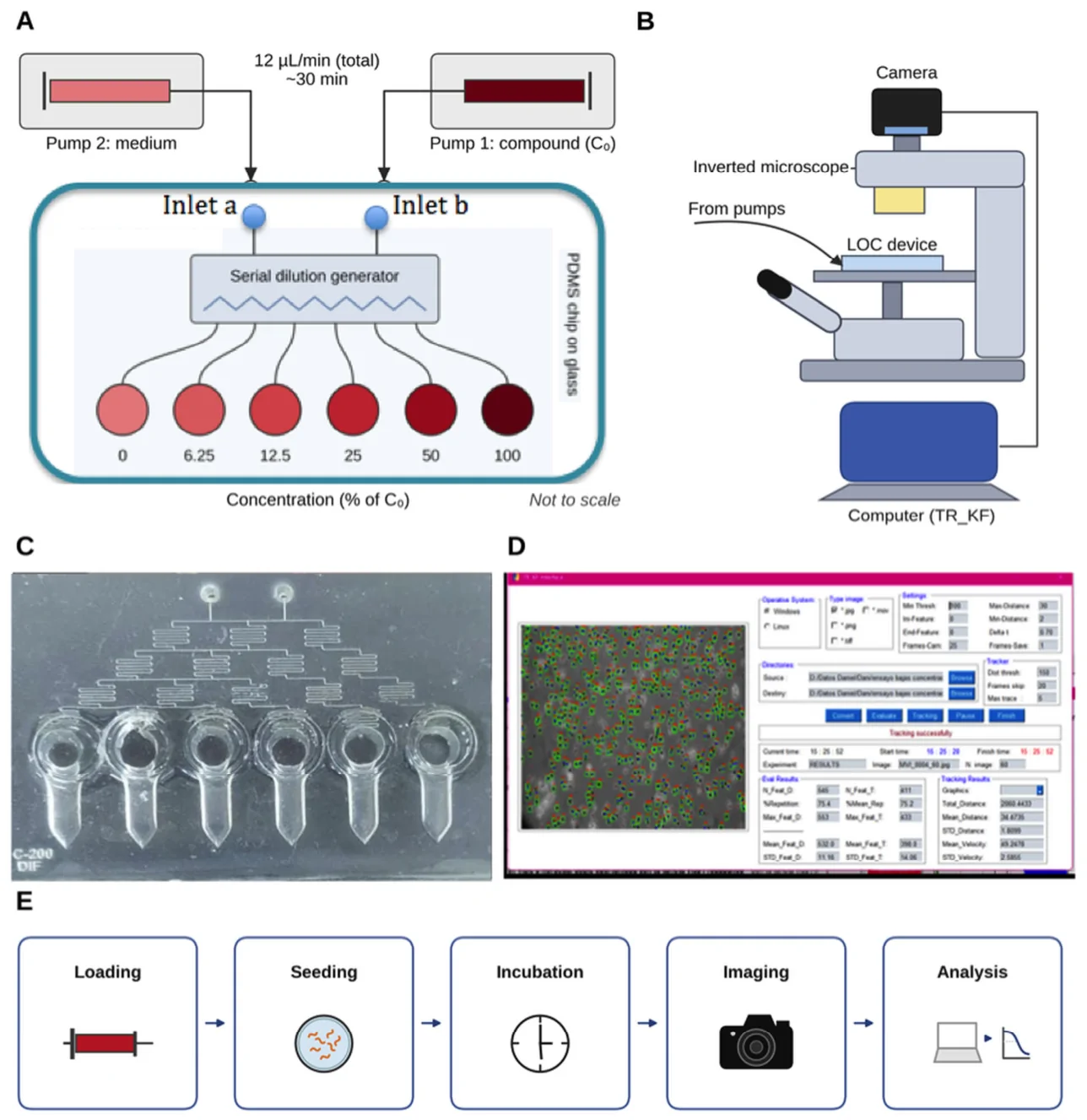
Experimental setup and workflow of the LOC–TR_KF platform for phenotypic screening of *T. cruzi* trypomastigotes. (**A**) Pump-driven serial dilution. Two syringe pumps were connected to the PDMS device, one supplying culture medium through inlet a and the other the compound solution at its highest concentration (C_0_) through inlet b. The pumps were operated at a combined flow rate of 12 µL/min for approximately 30 min. The serial dilution generator produces six concentrations (0 to 100% of C_0_), which are delivered to six outlet wells (5 mm in diameter) where the parasites were seeded. Red shading indicates the concentration of the compound in the culture medium. (**B**) Imaging setup. The device was placed on the stage of an inverted microscope, and videos were acquired with a camera and analyzed on a computer with TR_KF software (version 2.1.1). (**C**) Photograph of the LOC device showing the complete design with the inlets and the six outlet wells. (**D**) Representative video frame showing the TR_KF tracking overlay. (**E**) Experimental workflow: loading of the dilution series (~30 min); seeding of trypomastigotes (1 × 10^5^ per well); incubation for 24 h at 37 °C and 5% CO_2_; video acquisition (40× magnification; four videos per well, ~60 s each, 640 × 480 pixels, 25 fps); tracking of moving features with TR_KF software (mean number of tracked features per video, Mean_Feat_T); and nonlinear regression of the concentration–response curve, expressed relative to untreated controls, to obtain the IC_50_.

The microfluidic Lab-on-a-chip (LOC) device used in this study was previously described by Cadena Vizuete et al. [32]. The geometry of the device network remained unchanged; however, the inlets were enlarged from 1 mm [32] to 2 mm in diameter to connect the tubing of the syringe micropumps (Active A22; ADOX, Buenos Aires, Argentina), and the outlet wells were enlarged from 2.51 mm [32] to 5 mm in diameter to accommodate suspended parasites. The device was operated with pump-driven delivery, with each inlet connected to a syringe pump (Figure 1), instead of the direct inlet loading used previously [32], and the experimental conditions were optimized for the evaluation of extracellular *T. cruzi* trypomastigotes. Briefly, the device consisted of a symmetric microfluidic network of narrow channels that promoted diffusive mixing of soluble reagents, generating six two-fold serial dilutions of a concentrated solution (0% to 100%). These dilutions were delivered to six elongated culture chambers, each containing a circular outlet well (5 mm in diameter) for parasite seeding and incubation (Figure 1A,C).

The microfluidic devices were fabricated using the flexographic technique previously described [32,33,34], following these key steps: (a) design and UV-patterning of a photopolymer master mold (F-mold); (b) replication with epoxy resin to generate a reusable mold (ER-mold); (c) casting of polydimethylsiloxane (PDMS) (Sylgard 184, Dow Corning, Midland, MI, USA; 10:1 ratio) onto the ER-mold, followed by curing at 60 °C overnight and demolding; (d) perforation of the cured PDMS using biopsy punches (2 mm diameter) to generate the fluidic inlets (Integra Miltex^®^, Integra LifeSciences, Princeton, NJ, USA) for the culture medium and drug solutions, while a 5 mm diameter punches were employed to form wells for parasite seeding; (e) irreversible bonding of the PDMS layer to a glass slide by oxygen plasma treatment (BD-10AS, Electro-Technic Products, Inc., Chicago, IL, USA) for 120 s.

PDMS is the material routinely used in our laboratory for the fabrication of microfluidic devices for cell culture and bioproduction [32,35,36], and keeping the same material as in the device previously validated for intracellular amastigotes [32] allows direct comparison of the results obtained with both platforms. PDMS was also selected because of its optical transparency, gas permeability, and biocompatibility, and because its compatibility with soft lithography enables rapid, in-house fabrication and customization of microchannel geometries according to specific experimental requirements and with multiple reproducible replicas obtained from a single reusable mold [33,34,37,38]. These characteristics are particularly suitable for the parallel analysis of multiple concentration conditions while maintaining compatibility with microscopy-based image acquisition. A recognized limitation of PDMS is its capacity to absorb small hydrophobic molecules [39,40], which may affect the effective concentration of some compounds within microfluidic devices; this limitation and its implications for the present study are addressed in the Discussion.

### 2.2. Parasites and Cell Culture

Vero cells (a non-phagocytic cell line derived from African green monkey kidney cells) were maintained in RPMI-1640 (Invitrogen, Thermo Fisher Scientific, Waltham, MA, USA) supplemented with 5% fetal bovine serum (FBS; Internegocios S.A., Mercedes, Buenos Aires, Argentina) and 1% penicillin/streptomycin (Sigma-Aldrich, St. Louis, MO, USA) at 37 °C and 5% CO_2_.

*T. cruzi* trypomastigotes from the K98 strain (derived from the CA-1 strain, DTU TcI) [41,42,43] were obtained from Vero cell cultures infected at a multiplicity of infection (MOI) of 5:1 (trypomastigotes:cells). Trypomastigotes were harvested from the cell supernatants 5 days post-infection and maintained at 37 °C, 5% CO_2_. The MOI was calculated based exclusively on motile trypomastigotes observed at the time of harvest, consistent with the motility-based phenotypic criterion used throughout this study.

### 2.3. Compounds

Benznidazole (BZN) (Sigma-Aldrich, Cat. No 41966) was dissolved in PBS to prepare a 50 µM stock solution. The selection of candidate compounds was based on their previously reported activity against a broad range of pathogens, including ESKAPE bacteria [44,45] and protozoan parasites such as *Plasmodium* spp. [44] and *Leishmania donovani* [45,46]. In addition, previously determined IC_50_ values against intracellular *T. cruzi* amastigotes with the same candidates [32] were considered for comparative analysis in the present study. The compounds were provided in lyophilized form and reconstituted in dimethyl sulfoxide (DMSO, Sigma-Aldrich) in light-protected vials to obtain 10 mM stock solutions. These stock solutions were further diluted in RPMI-1640 culture medium to the working concentrations used in the assays.

The evaluated panel consisted of chemically synthesized small molecules and combinatorial libraries. The **2273** series comprised individual small molecules sharing a bis-guanidine core scaffold, with structural diversity generated by different combinations of substituent groups. In contrast, **2160-X** and **2161-X** correspond to mixture-based combinatorial libraries, in which multiple related molecules are present within each mixture. Each individual component within the mixtures is represented at approximately equimolar proportions. Specifically, the **2160-X** library contains molecules based on a bis-piperazine scaffold, whereas the **2161-X** library consists of molecules sharing spermine-like polyamine scaffold bearing bisguanidine functionalities, with diversity introduced through different combinations of R functional groups. Detailed information regarding the scaffolds, and functional groups represented in the **2160-X** and **2161-X** libraries has been previously reported [32].

### 2.4. LOC–TR_KF Platform

#### 2.4.1. LOC-Based Trypomastigote Motility Assay

Microfluidic devices were sterilized by autoclaving, rinsed sequentially with ethanol and sterile distilled water, and loaded with RPMI-1640 culture medium and compound solutions using two syringe pumps (Active A22; ADOX), one connected to each inlet (“a” to culture medium and “b” to compound solution). Both pumps were operated at 6 µL/min for approximately 30 min (combined flow rate, 12 µL/min; approximately 360 µL delivered in total), generating six concentration conditions through the serial dilution network of the device, as previously described [32]. Compound solutions introduced through inlet b were prepared taking into account the subsequent addition of the parasite suspension to the outlet wells, so that the intended final compound concentrations were achieved after parasite addition.

Compounds were prepared as 10 mM stock solutions in DMSO. The final DMSO concentration in all assays did not exceed 0.5% (*v*/*v*), a concentration previously reported to be compatible with *T. cruzi* viability assays without affecting compound activity [10]. Vehicle controls containing the corresponding DMSO concentration were included in all experiments, and no solvent-related effects on parasite viability were observed.

After filling the device, *T. cruzi* trypomastigotes were seeded into the outlet wells (2 × 10^6^ parasites/mL; 50 µL/well) and incubated for 24 h at 37 °C and 5% CO_2_, consistent with standard trypomastigote-based drug screening protocols that limit compound exposure to 24 h because parasite viability declines beyond this time point [9,47,48]. Four video recordings were acquired from randomly selected fields of view in each well using a Canon Rebel T3i EOS 600D camera (Canon Inc., Tokyo, Japan) coupled to a Zeiss Primovert inverted microscope (Carl Zeiss Microscopy GmbH, Jena, Germany) at 40× magnification.

Videos were analyzed using the TR_KF software (Section 2.4.2) for automated parasite tracking and motility-based quantification [32]. The motility-inhibitory concentration (IC_50_) was defined as the compound concentration required to reduce the motility-based TR_KF readout by 50% relative to untreated controls. IC_50_ values were determined by nonlinear regression using the log (inhibitor) versus normalized response model implemented in GraphPad Prism version 8.0 for Windows (San Diego, CA, USA). For each independent experiment, Mean_Feat_T values were normalized to the corresponding untreated control, which was set to 100%. Since trypomastigotes are non-replicative forms, IC_50_ values reported throughout this study represent inhibition of parasite motility rather than inhibition of parasite growth.

The candidate compounds and combinatorial mixtures were each evaluated in three independent experiments (*n* = 3), with four video recordings acquired from randomly selected fields per well and concentration in each experiment. A separate sterile LOC device was used for each independent experiment. All experimental parameters were kept constant throughout the study: flow rate, 12 µL/min combined (30 min approximately); compound solution introduced through inlet b and RPMI-1640 medium through the opposite inlet; six final concentration levels following the dilution scheme generated by the LOC device [32]; parasite suspension, 2 × 10^6^ parasites/mL (50 µL added per outlet well); incubation, 24 h at 37 °C and 5% CO_2_; and video duration, ~60 s. The final concentration series after parasite addition was 0, 3.125, 6.25, 12.5, 25, and 50 µM for BZN and the individual compounds, whereas the corresponding series for **2160-X** and **2161-X** was 0, 0.239, 0.479, 0.958, 1.915, and 3.83 µM. TR_KF analysis was performed as described in Section 2.4.2, with Max-Distance (30 px), Min-Distance (2 px), Delta t (0.70, software default), Dist thresh (150), Frames skip (20), and Max trace (5) held constant across experiments. Min-Thresh was adjusted within the range of 80–120 for each experiment, and a minimum repeatability criterion of %Mean_Rep ≥ 70% was required [32].

#### 2.4.2. Automated Motility Analysis with TR_KF Software

Videos were analyzed with TR_KF, a graphical user interface that detects parasites in each frame by contrast-limited histogram equalization, threshold-based binarization, and morphological operations, and links the resulting features into trajectories using a Kalman filter [32]. The version used in this study was TR_KF 2.1.1 (commit f038b09; upgraded from version 1.2.5, available at https://github.com/RamiroIsaJ/TR_KF_Interface (accessed on 8 October 2026)). This version updates that previously described [32] in several respects, summarized in Table 1; in particular, it allows the acquisition frame rate to be specified through the Frames-Cam and Frames-Save parameters. These parameters are relevant because parasite displacement between consecutive frames depends on the acquisition frame rate, which in turn affects detection against the minimum-displacement criterion (Min-Distance). Videos were acquired at 25 frames per second (fps), a rate that allowed moving parasites to be reliably distinguished from those without detectable movement under the conditions used here. A video was accepted for analysis only when repeatability (%Mean_Rep) reached the 70% criterion established for this platform [32], adjusting Min-Thresh as needed (Section 2.4).

Non-linear regression for IC_50_ determination was performed independently of TR_KF, using GraphPad Prism version 8.0 for Windows (San Diego, CA, USA). TR_KF does not store results internally; instead, it writes an output file at the end of each analysis, keeping the tracking module focused on efficient, low-cost feature detection and allowing the resulting motility data to be independently verified or re-fitted.

Concentration–response curves and IC_50_ values were based on Mean_Feat_T, the mean number of moving features tracked per video, averaged over the four videos acquired per well and expressed as a percentage of the untreated control. TR_KF detects and tracks moving parasites within the analyzed field; parasites without detectable movement are therefore not included in the automated readout. Accordingly, Mean_Feat_T provides a relative motility-based measurement rather than an absolute count of the total parasite population in the well. This criterion is conceptually consistent with the conventional manual assay, in which motile trypomastigotes with preserved morphology are counted, whereas immotile, rounded, or morphologically altered forms are excluded. Because this endpoint reflects loss of movement rather than a direct measure of parasite death, its interpretation as a surrogate marker of viability is addressed in the Discussion.

### 2.5. Validation of the LOC–TR_KF Platform

BZN was used as the reference compound to validate both the automated TR_KF-based motility assay and the integrated LOC–TR_KF platform for *T. cruzi* trypomastigote drug screening. Two complementary validation experiments were independently performed under comparable parasite densities, BZN concentration ranges, culture conditions, and incubation times.

In the first set of experiments, trypomastigotes (1 × 10^5^ parasites per condition) were incubated for 24 h in RPMI-1640 in 96-well plates. Parasite motility was independently quantified by manual counting of motile trypomastigotes with preserved morphology in a Neubauer chamber (reference method) and by automated analysis with TR_KF software, using videos acquired directly from the same wells. For automated analysis, four videos from randomly selected fields were acquired per BZN concentration and averaged to obtain a single value for each concentration within each experiment. For manual quantification, parasites were counted in four Neubauer chamber quadrants per BZN concentration, and the resulting counts were averaged to obtain a single value per concentration within each experiment. Four independent experiments were performed for both quantification methods (*n* = 4). Values obtained with each method were independently normalized to the corresponding untreated control, which was set to 100%. Concentration–response curves were compared using an extra sum-of-squares F-test in GraphPad Prism 8.0, which tested whether the datasets were adequately described by a single shared concentration–response curve or whether separate curves provided a significantly better fit (*p* < 0.05). IC_50_ values were estimated by nonlinear regression for each method and interpreted in the context of the corresponding concentration–response curves.

In the second set of experiments, the same BZN concentration range was evaluated using the LOC–TR_KF platform under the conditions previously described, including pump-driven delivery of the dilution series and parasite quantification with TR_KF software after 24 h of incubation. The concentration–response relationship obtained with the LOC–TR_KF platform was compared with that generated by the conventional reference assay (96-well plate followed by manual Neubauer counting) using the same statistical approach. The main operational characteristics of the LOC–TR_KF and conventional workflows, including assay-solution requirements, preparation of the concentration series, parasite quantification, and operator involvement, are summarized in Table 2.

The same parasite number (~1 × 10^5^ parasites per condition) was used in both assay formats. TR_KF tracking parameters were held constant across formats, as described in Section 2.4.2. Four independent LOC experiments were performed using four different microfluidic devices (one device per experiment; *n* = 4). For each BZN concentration, four videos from randomly selected fields were acquired per device and analyzed independently with TR_KF software. The four measurements were averaged to obtain a single value for each concentration and device.

To quantitatively assess assay reproducibility, intra-assay variability was evaluated by calculating the coefficient of variation (CV) among the four field measurements obtained for each condition within each LOC device. CVs were not calculated for conditions in which no motile parasites were detected by TR_KF software in any of the four analyzed fields. Chip-to-chip reproducibility was evaluated by independently fitting a BZN concentration–response curve for each of the four LOC devices and calculating the CV of the resulting IC_50_ values. CV was calculated as SD/mean × 100.

Because manual counting and TR_KF software generate different absolute quantitative readouts, comparisons between the methods were based on normalized concentration–response relationships and IC_50_ values rather than absolute parasite counts.

### 2.6. Infection Assay with Pre-Treated Trypomastigotes

To evaluate the effect of compound exposure on parasite infectivity, trypomastigotes (1 × 10^5^ per well) were pre-incubated for 24 h in 96-well plates with a single concentration of BZN, **2160-X**, or **2161-X**, corresponding to the IC_50_ previously determined by the LOC–TR_KF platform (see the results section), or maintained without treatment as a control. Following incubation, the pre-treated parasite suspension was diluted to achieve a multiplicity of infection (MOI) of 5 and used directly to infect Vero cell monolayers, without prior separation of motile and non-motile forms; the infective inoculum therefore consisted of the entire parasite population after treatment exposure, at the density required for the intended MOI.

Vero cells were seeded at 5 × 10^3^ cells/well in 96-well plates and infected with the diluted parasite suspension described above (MOI = 5). After 24 h of infection, non-internalized parasites were removed and cultures were maintained for 7 days. Cells were then fixed with methanol, stained with Giemsa (1:10), and analyzed microscopically (500 cells per condition) to determine infection rates, expressed as the percentage of infected cells [43]. Four independent experiments were performed for each experimental condition (*n* = 4), with 500 cells analyzed per condition in each experiment.

### 2.7. Cytotoxicity Assay

The cytotoxicity of the compounds toward Vero cells was evaluated using the 3-(4,5-dimethylthiazol-2-yl)-2,5-diphenyltetrazolium bromide (MTT, Sigma-Aldrich) colorimetric assay [49]. Cells (104 cells/well) were seeded in 96-well plates, left to adhere and exposed to serial dilutions of each compound for 48 h. These solutions were prepared as a five-point, two-fold serial dilution in culture medium, yielding final assay concentrations starting at 100 µM. After treatment, MTT (5 mg/mL) was added and cells were incubated for 2–4 h until formazan crystals formed. The crystals were then solubilized in isopropanol, and absorbance was measured at 570 nm.

Cell viability was expressed relative to untreated cells (100% viability) and cells treated with 1% Triton X-100 (Sigma-Aldrich; 0% viability). The half-maximal cytotoxic concentration (CC_50_) was calculated by nonlinear regression analysis using GraphPad Prism v8.0. For compounds that did not reduce cell viability by 50% at the highest concentration tested, CC_50_ values were reported as >100 µM. CC_50_ values are expressed as mean ± SD from three independent experiments (*n* = 3).

The selectivity index (SI) was calculated as the ratio between CC_50_ and the corresponding parasite IC_50_ value (SI = CC_50_/IC_50_). For amastigotes, IC_50_ values were obtained from our previously published study, which used the same compounds, LOC devices, and tracking software [32], differing only in parasite stage. In the present work, trypomastigote IC_50_ values were newly generated, and SI values for trypomastigotes were calculated accordingly. For SI calculations, values were considered not determined (n.d.) when either CC_50_ or IC_50_ exceeded the highest concentration tested.

## 3. Results

### 3.1. Validation of the Automated Quantification Method and the LOC–TR_KF Platform

**Figure 2:**
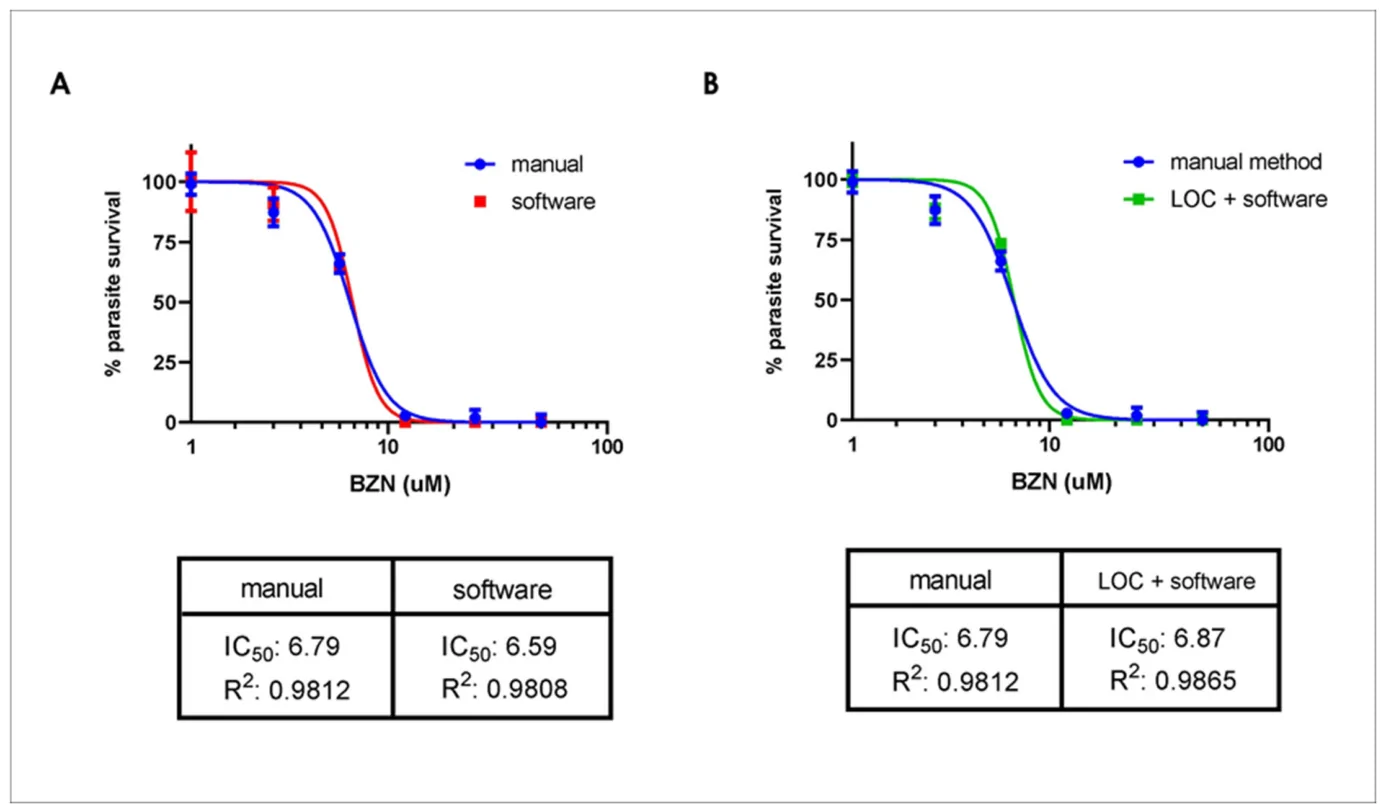
Validation of the automated quantification method and the integrated LOC platform using benznidazole (BZN). (**A**) Dose–response curves obtained for BZN against *T. cruzi* trypomastigotes in the conventional 96-well assay using manual Neubauer counting (blue) and automated motility quantification with the TR_KF software (red). Curve comparison was performed using the extra sum-of-squares F-test, which compares the fits of two nested models by least-squares regression (*p* = 0.65, F = 0.43). (**B**) Dose–response curves obtained for BZN using the conventional reference assay (96-well plate followed by manual Neubauer counting; blue) and the integrated LOC–TR_KF platform (green). Curve comparison was performed using the extra sum-of-squares F-test (*p* = 0.09, F = 2.54). Dose–response curves were generated from four independent experiments (*n* = 4). For TR_KF software analysis, four videos from randomly selected fields per concentration were averaged within each experiment. Four different LOC devices were independently evaluated in these experiments. IC_50_ and R^2^ values are shown in the corresponding graphs.

To validate the automated quantification method (TR_KF software) and the integrated LOC–TR_KF platform, the performance of the motility assay was evaluated using BZN as the reference drug. First, trypomastigote responses to BZN were evaluated in conventional 96-well plates using two complementary quantification methods: (i) manual microscopy based on parasite motility and morphological integrity, and (ii) automated motility tracking using the TR_KF software. Manual evaluation relied on the identification of motile parasites displaying preserved morphology, whereas automated analysis detected and tracked motile parasites directly from video recordings. Both approaches generated highly similar concentration–response curves, yielding IC_50_ values of 6.79 µM (R^2^ = 0.9812) and 6.59 µM (R^2^ = 0.9808) for manual and automated quantification, respectively. Comparison of the fitted curves using an extra sum-of-squares F test revealed no significant differences between methods (F = 0.43, *p* = 0.65), indicating that both datasets were adequately described by a common concentration–response curve (Figure 2A).

The complete LOC–TR_KF platform was then evaluated by comparing its concentration–response curve and IC_50_ value with those obtained using the conventional reference assay (96-well plate + manual Neubauer counting). The LOC–TR_KF platform yielded an IC_50_ of 6.87 µM, closely matching the value obtained with the conventional reference assay (6.79 µM). Comparison of the fitted concentration–response curves using the extra sum-of-squares F-test revealed no significant difference between methods (F = 2.54, *p* = 0.09), indicating that both datasets were adequately described by a common concentration–response curve (Figure 2B).

Reproducibility of the LOC–TR_KF platform was further assessed across four independently tested LOC devices. Within each device, variability among the four analyzed fields in each well was low for conditions in which motile parasites were detected, with intra-assay CVs ranging from 1.6% to 4.5% (mean 3.16 ± 0.95%). At 12, 25, and 50 µM BZN, no motile parasites were detected by TR_KF software in any of the four analyzed fields, and CVs were therefore not calculated. Independent concentration–response fitting for the four LOC devices yielded IC_50_ values of 6.896, 6.698, 6.912, and 6.848 µM (mean 6.84 ± 0.10 µM), corresponding to an inter-chip CV of 1.43%. These results indicate low within-assay and chip-to-chip variability under the experimental conditions evaluated.

Together, these findings support the use of the integrated LOC–TR_KF platform as a reliable approach for determining concentration–response relationships and IC_50_ values in trypomastigote drug screening while enabling automated, objective, and less labor-intensive parasite quantification.

### 3.2. Screening of Candidate Compounds Against T. cruzi Trypomastigotes in the LOC–TR_KF Platform

The evaluated panel comprised two combinatorial libraries (**2160-X** and **2161-X**) and eight individual compounds from the **2273** series, all previously evaluated in an intracellular *T. cruzi* assay targeting the amastigote stage [32]. In that study, antiparasitic activity against intracellular amastigotes was indirectly assessed by quantifying the release of trypomastigotes from infected Vero cells following drug treatment, which served as a surrogate measure of intracellular parasite development. The IC_50_ values obtained here against extracellular trypomastigotes, together with the previously reported IC_50_ values for the intracellular amastigote assay, the CC_50_ values determined in Vero cells, and the corresponding selectivity indices (SI), are summarized in Table 3.

Among the compounds evaluated, the reference drug BZN exhibited an IC_50_ value of 6.9 µM against trypomastigotes, a CC_50_ of 113.1 µM in Vero cells, and a SI of 16.4. Similarly, compounds **2161-X**, **2160-X**, and **2273-2** exhibited IC_50_ values in the low micromolar range and SI values above 10. The **2161-X** library showed the highest calculated SI for the trypomastigote assay (SI = 130.5), followed by **2160-X** (SI = 12.5) and **2273-2** (SI = 13.1), with selectivity values comparable to BZN. In contrast, compound **2273-18** showed high cytotoxicity (CC_50_ < 10 µM), whereas the remaining compounds exhibited low selectivity (SI < 7).

Comparison of these parameters allowed a preliminary assessment of compound activity against trypomastigotes in relation to host-cell cytotoxicity and previously reported activity against amastigotes. It should be noted that **2160-X** and **2161-X** correspond to mixture-based combinatorial libraries rather than individual compounds. Consequently, the IC_50_ and SI values reported for these samples represent the integrated activity of the corresponding chemical libraries and cannot be attributed to a single molecular entity.

The selectivity profiles differed when evaluated against the amastigote stage. While BZN showed favorable selectivity in amastigotes (SI = 35.3), compounds **2161-X** and **2160-X** exhibited substantially lower SI values (0.7 and 1.7, respectively). These findings highlight marked stage-dependent differences in compound activity.

### 3.3. Effect of Compound Pre-Treatment on the Infectivity of T. cruzi Trypomastigotes

**Figure 3:**
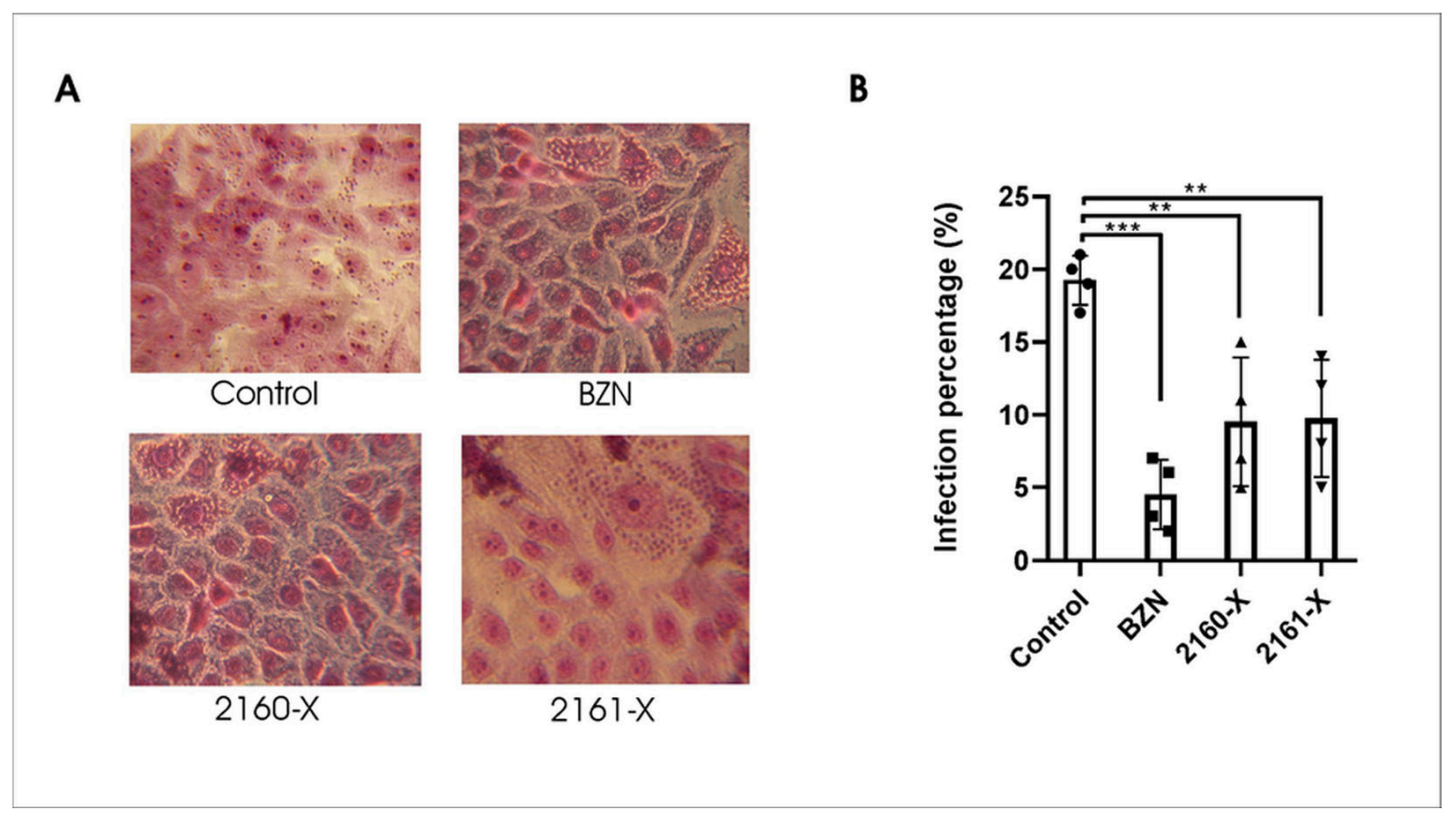
Effect of compound pre-treatment on the infectivity of *T. cruzi* trypomastigotes. Parasites were pre-treated for 24 h in 96-well plates with BZN, **2160-X**, or **2161-X** at their respective IC_50_ concentrations, previously determined using the LOC–TR_KF platform. Following pre-treatment, trypomastigotes were used to infect Vero cell monolayers in 96-well plates. Untreated parasites were used as controls. Infection rates were determined 7 days post-infection by staining and optical microscopy. (**A**) Representative microscopy images of Vero cell cultures infected with untreated trypomastigotes or with trypomastigotes pre-treated with BZN, **2160-X**, or **2161-X**. (**B**) Percentage of infected Vero cells under each experimental condition. Data are representative of one of four independent biological experiments, which showed similar trends. Bars represent the mean ± SD, and each symbol represents an individual data point; different symbol shapes are used only to distinguish the experimental conditions. ** *p* < 0.01; *** *p* < 0.001 compared with untreated control.

Trypomastigotes were pre-treated for 24 h in 96-well plates with the respective IC_50_ concentrations of the selected compounds (Table 3) and subsequently used to infect Vero cell monolayers cultured in 96-well plates. Infection rates were determined 7 days post-infection. Results show that untreated parasites infected 19,2% of host cells, whereas pre-treatment with BZN reduced the infection rate to 4.5%. Parasites pre-treated with **2160-X** and **2161-X** produced infection rates of 9.5% and 9.7%, respectively (Figure 3). Both experimental compounds significantly reduced host-cell infection levels compared with untreated controls, corresponding to an approximately 50% reduction in infection rate. Although this effect was less pronounced than that of BZN, the results indicate that pre-treatment with **2160-X** and **2161-X** was associated with a reduced capacity of the parasite population to establish infection under the experimental conditions evaluated.

## 4. Discussion

Here, we report the validation and application of an integrated Lab-on-a-Chip (LOC) microfluidic platform coupled with automated motility tracking (TR_KF software) to screen candidate compounds against *Trypanosoma cruzi* trypomastigotes. The development of new chemotherapeutic options for Chagas disease remains a critical global health need, given the limited efficacy and toxicity of current treatments, the complexity of this parasite’s life cycle, and the disease’s substantial social and economic impact [2].

The first validation step, using benznidazole (BZN) as the reference drug in conventional 96-well plates, demonstrated that automated motility quantification produced concentration–response curves and IC_50_ values comparable to those obtained by conventional manual counting in a Neubauer chamber. By reducing reliance on manual parasite counting and providing a permanent video record that can be re-analyzed, automated motility tracking retains a biologically relevant phenotypic readout, given that trypomastigote motility plays an important role in host-cell invasion, tissue dissemination and infection establishment [50]. The 24 h exposure period used throughout this study is consistent with previous studies showing that the effects of nitroheterocyclic drugs such as BZN and nifurtimox on non-replicative *T. cruzi* trypomastigotes can be detected within this time frame [47,48]. This relatively short exposure period is particularly suitable for trypomastigote-based assays, in which prolonged incubation may be limited by the progressive loss of parasite viability under in vitro culture conditions. Consequently, this image-based approach provides an automated method for phenotypic screening of compounds affecting trypomastigote motility.

The second validation step demonstrated that the complete LOC–TR_KF platform generated concentration–response relationships and IC_50_ values comparable to those generated by the conventional reference assay. The absence of significant differences between the concentration–response curves supports the ability of the complete LOC–TR_KF platform to reproduce the BZN pharmacological response obtained with the conventional assay under the conditions evaluated. The low within-assay and inter-LOC variability observed across independent experiments further supports the reproducibility of the microfluidic workflow.

Importantly, reproducing the pharmacological response obtained in conventional plates is not, by itself, the principal advantage of the LOC format. Rather, the microfluidic device generates six concentration conditions from two inlet solutions within a single device, reducing the volume of assay solutions required and the manual handling associated with the preparation of multiple concentration conditions. As summarized in Table 2, these features, together with automated video-based analysis, streamline the experimental workflow compared with conventional plate-based preparation and manual Neubauer counting. In addition, the video recordings provide a permanent experimental record that can be re-analyzed if required. Thus, the added value of the LOC–TR_KF platform lies in combining miniaturized generation of concentration conditions with automated, label-free phenotypic analysis rather than in producing a pharmacological response different from that of the conventional assay. Recent studies have described bioluminescent or reporter-based screening platforms for *T. cruzi* [13,14,15,16]. Our approach complements these technologies by combining microfluidics with label-free automated image analysis without requiring reporter-expressing parasite lines.

An inherent limitation of PDMS-based microfluidic platforms is the potential absorption of small hydrophobic molecules, which may reduce their effective concentrations within the device during testing [39,40,51]. PDMS was nevertheless selected for its optical clarity, biocompatibility, and suitability for in-house device fabrication [32,33,34,38], as well as to maintain methodological continuity with our previously validated LOC platform. Alternative materials with lower small-molecule absorption, including thermoplastics and other polymeric materials, may mitigate this limitation, although their implementation requires adaptation of the fabrication and bonding procedures [51,52]. The close agreement between the IC_50_ values obtained for BZN in the LOC device (6.87 μM) and the conventional 96-well assay (6.79 μM) suggests that any potential PDMS-related loss of BZN did not have a substantial impact on the concentration–response relationship under the experimental conditions used here. However, compound absorption was not directly measured, and compound-dependent effects cannot be excluded, particularly for more hydrophobic molecules. Evaluating compound recovery and/or alternative device materials should therefore be considered in future studies.

Application of the validated platform identified the mixture-based combinatorial libraries **2161-X** and **2160-X** as the most active samples against trypomastigotes, with IC_50_ values of 0.2 and 1.1 μM, respectively. Among the evaluated samples, **2161-X** showed the highest SI against trypomastigotes (130.5), markedly higher than that of BZN (16.4), reflecting its high activity against this parasite stage relative to its cytotoxicity in Vero cells. However, this high selectivity was strongly stage-dependent. Indeed, comparison with previously reported intracellular amastigote data [32] revealed a markedly different profile: the SI of **2161-X** decreased from 130.5 against trypomastigotes to 0.7 against amastigotes, whereas BZN showed favorable selectivity against both stages (SI = 16.4 and 35.3, respectively). These findings highlight the marked stage-dependent differences in compound susceptibility and illustrate the importance of evaluating candidate compounds against biologically distinct stages of *T. cruzi*. Extracellular trypomastigotes and intracellular amastigotes differ in their biological state and drug exposure environment. While trypomastigotes are directly exposed to compounds in the culture medium, activity against intracellular amastigotes additionally depends on compound access to the host cell and intracellular parasite. These differences may contribute to the distinct potency and selectivity profiles observed between parasite stages and support the use of stage-specific assays as complementary tools for compound characterization. Current *T. cruzi* drug discovery strategies likewise incorporate assays addressing different parasite stages and pharmacodynamic properties as complementary components of compound characterization [9,47,48,53,54]. An inherent limitation of **2161-X** and **2160-X** is their nature as complex combinatorial mixtures; further chemical deconvolution will be necessary to identify and characterize the individual constituents responsible for the observed activity.

Regarding functional readouts, the infection assay showed that 24 h pre-treatment with **2160-X** and **2161-X** at their respective IC_50_ concentrations reduced host-cell infection rates by approximately 50%, compared with a larger reduction following BZN exposure. Because the complete pre-treated parasite suspension was used for infection, this endpoint reflects the combined effect of compound exposure on parasite motility and overall infective population density, rather than an isolated measure of host-cell invasion efficiency alone. In this sense, motility patterns in *T. cruzi* trypomastigotes have been shown to correlate with host-cell invasion efficiency [50], supporting the biological relevance of motility as a phenotypic readout for this parasite stage.

Nevertheless, an important limitation of the present approach is that TR_KF software quantifies detectable parasite movement rather than viability per se. Parasites without detectable movement are not included in the automated readout; consequently, loss of detectable motility cannot be interpreted as definitive evidence of parasite death. This limitation also applies, to some extent, to the conventional microscopic assay used for comparison, in which motile trypomastigotes with preserved morphology are counted, whereas immotile, rounded, or morphologically altered forms are excluded. Importantly, TR_KF-based motility measurements were not validated against an independent parasite viability assay in the present study. Accordingly, the LOC–TR_KF platform should be regarded as a motility-based phenotypic screening approach for identifying compounds that markedly affect trypomastigote motility, rather than as a direct assay of trypanocidal activity. Selected hits could subsequently be evaluated using independent viability readouts to determine whether loss of detectable movement is associated with parasite death. For prioritized compounds, washout/recovery experiments could additionally help distinguish reversible motility inhibition from irreversible effects. The infection assay performed here provides complementary functional information for selected compounds but does not substitute for an independent viability assay.

Future developments could incorporate advanced computer-vision approaches to extract additional dynamic features from parasite trajectories and identify motility phenotypes beyond the current Mean_Feat_T endpoint. Such features would require independent biological validation before specific movement patterns could be associated with parasite viability or particular drug responses.

In conclusion, the present study validates both the automated TR_KF motility quantification method and its integration within a LOC platform for phenotypic screening of *T. cruzi* trypomastigotes. The LOC-TR_KF platform generated BZN concentration–response relationships and IC_50_ values comparable to those obtained with the conventional assay, with low within-assay and inter-device variability, while reducing assay-solution requirements and manual parasite-counting steps and providing a permanent video record for subsequent re-analysis. Together with our previous validation for intracellular amastigotes [32], these findings extend the application of the LOC–TR_KF platform to extracellular trypomastigotes and support its use as a complementary motility-based phenotypic screening tool for stage-specific characterization of anti-*T. cruzi* compounds.

## Figures and Tables

**Table 1:** Comparison between the TR_KF software as originally described [32] and the version (2.1.1) used in this study.

Feature	Original TR_KF [32]	Updated TR_KF (This Study)
Video input format	Image sequences (.jpg, .png, .tiff)	Adds direct import of video files (.mov) via a new conversion module, prior to feature extraction
Acquisition rate calibration	Not adjustable; a fixed acquisition rate was assumed	Frames-Cam and Frames-Save controls added, so the acquisition and frame-saving rates can be set to match the video’s actual recording rate
Minimum inter-frame displacement for tracking (Min-Distance)	5 px (example dataset)	2 px, recalibrated for the 25 fps acquisition rate used in this study
Repeatability criterion	≥70%, based on the total number of detections across the dataset	≥70%, with a short-term index (%Repetition) computed over the first nine frames, in addition to the dataset-wide mean (%Mean_Rep)
Preprocessing filter	Median filter, for noise reduction	Images were contrast-equalized via CLAHE (10×10 contextual window) and subsequently evaluated using the structural similarity index (SSIM)
Binarization method	Otsu threshold	Manual Min-Thresh (range 80–120), adjusted per experiment

**Table 2:** Operational comparison of the LOC-TR_KF platform and the conventional method.

Parameter	LOC-TR-KF Platform	Conventional Method (96-Well Plate + Neubauer Counting)
Concentration conditions per assay	6	6
Generation of concentration series	Pump-driven within the microfluidic device from two inlet solutions	Manual serial dilution
Assay solution volume for six concentration conditions (a)	~360 µL delivered through the two LOC inlets	150 µL dispensed per condition (~900 µL for six conditions)
Parasite suspension added	50 µL per condition	50 µL per condition
Preparation of the dilution series	Automatic, pump-driven (~30 min)	Manual serial dilution
Parasite quantification	Automated (TR_KF software); four ~60 s videos per condition	Manual counting in a Neubauer chamber (four quadrants), ~5–7 min per well
Estimated hands-on operator time per compound (b)	~25 min (video recording)	~45–60 min (Neubauer chamber loading, cell counting, washing and reloading steps).
Data record	Video recordings available for subsequent re-analysis	Manual counts

(a) For the LOC–TR_KF platform, the reported volume corresponds to the total volume delivered through both inlets during generation of the six concentration conditions (6 µL/min per inlet for approximately 30 min). For the conventional assay, the reported volume corresponds to the compound dilutions dispensed into the six assay wells (150 µL per condition), excluding any additional volume required for preparation of the serial dilution. (b) Hands-on operator time refers to the approximate time requiring active operator intervention and does not include incubation periods or unattended pump operation.

**Table 3:** In vitro activity against *T. cruzi*, cytotoxicity in Vero cells, and selectivity indices of the evaluated compounds.

Compound	CC_50_ (µM)	IC_50_ Tryp (µM)	SI Tryp	IC_50_ Amas (µM)	SI Amas
**2273-2**	40.6 ± 20.5	3.1 ± 0.2	13.1	>50	n.d.
**2273-4**	31.1 ± 11.7	13.3 ± 0.4	2.3	13.2	2.4
**2273-7**	65.26 ± 54.20	10.2 ± 0.5	6.4	>50	n.d.
**2273-9**	35.4 ± 11.8	15.2 ± 0.4	2.3	>50	n.d.
**2273-15**	>100	21.6 ± 0.5	n.d.	>50	n.d.
**2273-18**	8.3 ± 0.2	10.3 ± 0.3	0.8	>50	n.d.
**2273-19**	>100	20.5 ± 0.5	n.d.	>50	n.d.
**2273-21**	29.8 ± 9.8	21.7 ± 0.2	1.4	>50	n.d.
**2160-X**	13.8 ± 1.1	1.1 ± 0.0 *	12.5	8.1	1.7
**2161-X**	26.1 ± 0.7	0.2 ± 0.0 *	130.5	39.3	0.7
BZN	113.1 ± 23.4	6.9 ± 0.2	16.4	3.2	35.3

CC_50_ values were determined in Vero cells in the present study and IC_50_ values for trypomastigotes were determined using the LOC–TR_KF platform described herein, whereas IC_50_ values for intracellular amastigotes were previously reported by Cadena Vizuete et al. [32]. CC_50_ and IC_50_ values for trypomastigotes are expressed as mean ± SD from three independent experiments (*n* = 3). For LOC-based trypomastigote assays, a separate LOC device was used for each independent experiment, and four video recordings per well and concentration were averaged within each experiment. Selectivity indices (SI) were calculated as CC_50_/IC_50_. When CC_50_ or IC_50_ values exceeded the highest concentration tested, SI was not determined. The highest final concentration tested was 50 µM for BZN and the individual compounds, and 3.83 µM for the mixture-based combinatorial libraries **2160-X** and **2161-X** (marked with an asterisk). Tryp, trypomastigotes; amas, amastigotes; CC_50_, 50% cytotoxic concentration; IC_50_, 50% inhibitory concentration; SI, selectivity index; n.d., not determined; BZN, benznidazole.

## Data Availability

Data presented in this study are available from the corresponding authors upon request.

## Abbreviations

The following abbreviations are used in this manuscript:

BZN	benznidazole
CC_50_	half-maximal cytotoxic concentration
DMSO	dimethyl sulfoxide
IC_50_	half-maximal inhibitory concentration
LOC	Lab-on-a-chip
MOI	multiplicity of infection
MTT	3-(4,5-dimethylthiazol-2-yl)-2,5-diphenyltetrazolium bromide
PDMS	polydimethylsiloxane
SI	selectivity index
